# Advances in Enantioselective Synthesis and Chiral Resolution of Insecticides

**DOI:** 10.3390/molecules31101667

**Published:** 2026-05-15

**Authors:** Carlos Alberto López-Rosas, Enrique Delgado-Alvarado, Felipe Barrera-Méndez, Israel Bonilla-Landa, José Luis Olivares-Romero

**Affiliations:** 1 Red de Estudios Moleculares Avanzados, Instituto de Ecología, Campus III, Carretera Antigua a Coatepec 351, Col. El Haya, Xalapa 91000, Veracruz, Mexico; carlos.lopez@inecol.mx (C.A.L.-R.); felipe.barrera@inecol.mx (F.B.-M.); israel.bonilla@inecol.mx (I.B.-L.); 2 Micro and Nanotechnology Research Center, Universidad Veracruzana, Blv. Av. Ruiz Cortines No. 455 Fracc. Costa Verde, Boca del Río 94294, Veracruz, Mexico; endelgado@uv.mx; 3 Investigador por México SECIHTI, Avenida Insurgentes Sur 1582, Colonia Crédito Constructor, Demarcación Territorial Benito Juárez, Ciudad de México 03940, Mexico

**Keywords:** enantioselective, chirality, insecticides, chiral resolution

## Abstract

Chirality has emerged as a critical determinant in the design, efficacy, and environmental behavior of modern insecticides. While a significant proportion of agrochemicals are inherently chiral, most are still commercialized as racemic mixtures, despite well-documented differences in biological activity, toxicity, and degradation pathways between enantiomers. In this review, we provide a comprehensive and critical analysis of advances in the stereoselective synthesis and resolution of chiral insecticides, with particular emphasis on neonicotinoids, pyrethroids, and oxadiazines, including indoxacarb. A systematic survey of the literature (1985–2025), including peer-reviewed articles and patents, reveals that multiple strategies have been developed to access enantiomerically enriched compounds, including asymmetric organocatalysis, transition-metal catalysis, chiral-pool approaches, biocatalytic transformations, and chromatographic resolution techniques. Among these, recent developments in photoredox catalysis, recyclable metal complexes, and enzyme-mediated processes have significantly improved enantioselectivity and scalability, bridging the gap between academic methodologies and industrial applications. Despite these advances, challenges remain in achieving cost-effective, sustainable, and universally applicable asymmetric processes. Importantly, the relationship between stereochemistry and biological performance underscores the need for integrating synthetic chemistry with toxicological and environmental studies. Future directions point toward the incorporation of green chemistry principles, continuous-flow processes, and computational tools, including machine learning and molecular modeling, to accelerate the rational design of enantiopure agrochemicals. This review highlights both the progress achieved and the critical gaps that must be addressed to realize the potential of stereoselective insecticide development fully.

## 1. Introduction

Modern insecticide discovery stems from two contrasting historical trajectories: pyrethroids, derived from the natural pyrethrins found in chrysanthemum flowers, and neonicotinoids, inspired by the alkaloid nicotine. Natural pyrethrins have been used for centuries, but are photolabile. After their structures were elucidated in the 1940s and 1950s, chemists began to replicate and modify them. The first synthetic pyrethroid, allethrin, was invented by the USDA in 1949 and commercialized in 1953. Initially, its stereochemistry was not fully understood, illustrating how chirality in pyrethroids was overlooked during early development [1]. Subsequent generations of synthetic pyrethroids introduced electron-withdrawing substituents (e.g., halogens, cyano groups) to enhance potency and photostability; these modifications also introduced multiple stereogenic centers, giving rise to complex mixtures of diastereomers and enantiomers. Neonicotinoids were developed decades later. They mimic the insecticidal properties of nicotine but avoid its mammalian toxicity. Introduced commercially in the early 1990s, neonicotinoids quickly became the largest class of insecticides worldwide because of their systemic action and high selectivity for insect nicotinic acetylcholine receptors (nAChRs). Their pharmacophores fall into three subclasses: *N*-nitroguanidines (e.g., imidacloprid, thiamethoxam, clothianidin, dinotefuran), nitromethylenes (e.g., nitenpyram), and *N*-cyanoamidines (e.g., acetamiprid) [2]. The electron-rich nitromethylene, nitroimine, or cyanoimine group interacts with the insect nAChR to confer potency and selectivity [2]. Some recent compounds, such as cycloxaprid and paichongding, are *cis*-locked neonicotinoids, which means that the nitro or cyano substituent and its adjacent moiety adopt a *cis* orientation within a bicyclic framework [2]. This structural constraint improves binding affinity but generates stereogenic centers, making the compounds chiral.

Chirality is increasingly recognized as a critical factor in agrochemicals. Roughly 25% of pesticides currently in use are chiral [3], and enantiomers often behave as distinct compounds: they can vary dramatically in acute toxicity to aquatic invertebrates and undergo enantioselective degradation in soils and sediments [3,4]. Yet most chiral insecticides are marketed as racemic mixtures because separating or synthesizing single enantiomers is expensive [3]. In pyrethroids, for example, specific stereoisomers confer most of the biological activity; other isomers may be inactive or even harmful, emphasizing the need for stereoselective synthesis and resolution strategies [4]. Similarly, chiral neonicotinoids display differences in insecticidal potency and environmental fate across enantiomers, as seen with cycloxaprid and dinotefuran derivatives [4]. Regulatory pressures further motivate the move toward enantiomerically pure insecticides. Widespread neonicotinoid use has been implicated in pollinator declines. The European Food Safety Authority (EFSA) concluded in 2013 that imidacloprid, clothianidin, and thiamethoxam pose an unacceptably high risk to bees; the European Union subsequently restricted these compounds on flowering crops and later banned them for all outdoor uses [2]. Health Canada and the U.S. Environmental Protection Agency followed with restrictions or cancellations of certain uses, and several U.S. states have legislated neonicotinoid limits [2]. At the same time, insect pests have developed resistance to neonicotinoids and pyrethroids, prompting interest in new scaffolds with different binding modes and reduced propensity for cross-resistance. These historical developments, the stereochemical complexity of both pyrethroids and *cis*-locked neonicotinoids, and the growing awareness that enantiomers differ in toxicity and environmental persistence collectively underscore the importance of stereoselective design. The remainder of this review, therefore, focuses on recent advances in asymmetric synthesis, enzymatic resolution, and chromatographic separation of chiral insecticides, with an emphasis on cycloxaprid/paichongding, dinotefuran derivatives, pyrethroid analogues (cypermethrin, fenvalerate, cycloprothrin), and the oxadiazine indoxacarb. Despite notable advances, the field of stereoselective insecticide synthesis remains unevenly developed, with significant progress in catalytic methodologies but limited integration with biological evaluation and industrial implementation.

## 2. Literature Search Strategy

A rigorous literature search strategy underpins this review. We systematically surveyed major bibliographic databases, including PubMed, Web of Science, Scopus, ScienceDirect, and Google Scholar, as well as specialized chemical and patent resources such as SciFinder, Reaxys, Espacenet, and Google Patents. Searches were conducted for publications from 1985 through 2025, a time span chosen to encompass the discovery of early neonicotinoids and pyrethroids and the development of contemporary chiral catalysts. We combined keywords related to chirality (“chiral,” “enantiomer,” “enantioselective,” “asymmetric synthesis,” “chiral resolution”) with specific compound names (“cycloxaprid,” “paichongding,” “dinotefuran,” “cypermethrin,” “fenvalerate,” “cycloprothrin,” “indoxacarb,” etc.) and general class descriptors (“neonicotinoid,” “pyrethroid,” “oxadiazine”). Reference lists of relevant papers and reviews were also inspected to capture additional sources. Records were included if they were peer-reviewed articles or patents written in English that reported synthetic or resolution methods for chiral insecticides; we excluded grey literature, conference abstracts, non-English publications, and studies focused solely on environmental fate, toxicology, or formulation without synthetic detail. This approach follows general guidance that literature searches should be systematic and organized to identify high-quality references [5]. No primary data were collected; rather, all information was extracted directly from the selected literature to provide a transparent and reproducible foundation for this review.

## 3. Chiral Neonicotinoids

### 3.1. Paichongding

**Figure 1:**
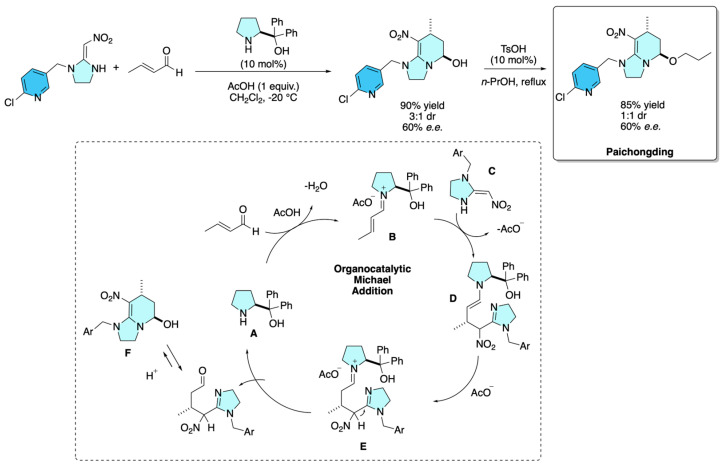
Organocatalytic synthesis of Paichongding.

Paichongding has been synthesized enantioselectively through an organocatalytic route reported by Lou and co-workers in 2015 [6]. The key step involves a diphenylprolinol trimethylsilyl ether-catalyzed Michael addition of 2-nitromethylene imidazolidines to α,β-unsaturated aldehydes under mild conditions, affording hexahydroimidazo[1,2-a]pyridine scaffolds in high yield (up to 90%) with excellent enantioselectivity (up to 95% *e.e.*) and good diastereoselectivity. Subsequent acetal-amination/etherification steps facilitated structural confirmation, with X-ray analysis establishing the (5*R*,7*R*) configuration. A representative Michael adduct was then smoothly converted to cycloxaprid in a single step by refluxing in *n*-propanol with catalytic *p*-toluenesulfonic acid, giving the insecticide in 85% yield while retaining enantioenrichment (ca. 60% *e.e.*). Importantly, the method proved scalable to gram quantities without loss of stereocontrol, highlighting the practicality of this asymmetric organocatalytic approach for accessing enantiopure neonicotinoids (Figure 1).

As depicted in Figure 1, the α,β-unsaturated aldehyde is initially activated by catalyst **A**, generating iminium intermediate **B**. Subsequently, the Michael donor **C** attacks the iminium species from the *Re* face, thereby minimizing steric repulsion with the diarylsilyl ether substituent. The resulting intermediate **D** then undergoes isomerization to intermediate **E**, which, upon hydrolysis, affords the desired product **F**, while simultaneously regenerating the catalyst.

**Figure 2:**
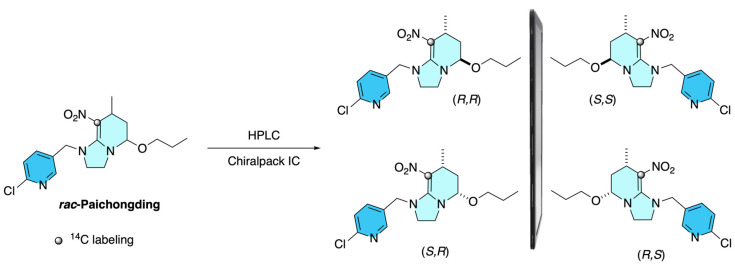
Preparative chiral HPLC (separation): Daicel Chiralpak IC column, 250 × 30 mm, 5 μm; isocratic elution with absolute ethanol; flow 20 mL·min^−1^; 35 ± 1 °C; UV detection at 325 nm; fractions manually collected.

Another report by Li in 2013 describes that the racemic ^14^C-labeled Paichongding was separated into its four stereoisomers by preparative chiral HPLC using a Daicel Chiralpak IC column under isocratic ethanol elution. Baseline resolution was achieved, and the individual fractions were collected and identified through retention time, optical rotation, and comparison with authentic non-labeled standards. Each stereoisomer was obtained with high chemical purity (>98%) and radiochemical purity (>96%), providing enantiomerically well-defined samples for subsequent environmental fate investigations (Figure 2) [7].

### 3.2. Cycloxaprid

To the best of our knowledge, despite the well-recognized presence of two stereogenic centers in cycloxaprid, no studies have reported the application of modern asymmetric strategies, such as organocatalysis or metal-mediated enantioselective transformations, to construct the oxabridged *cis*-nitromethylene scaffold characteristic of this molecule. Consequently, the development of catalytic enantioselective approaches to cycloxaprid represents a significant, largely unexplored opportunity for innovation in agrochemical design.

**Figure 3:**
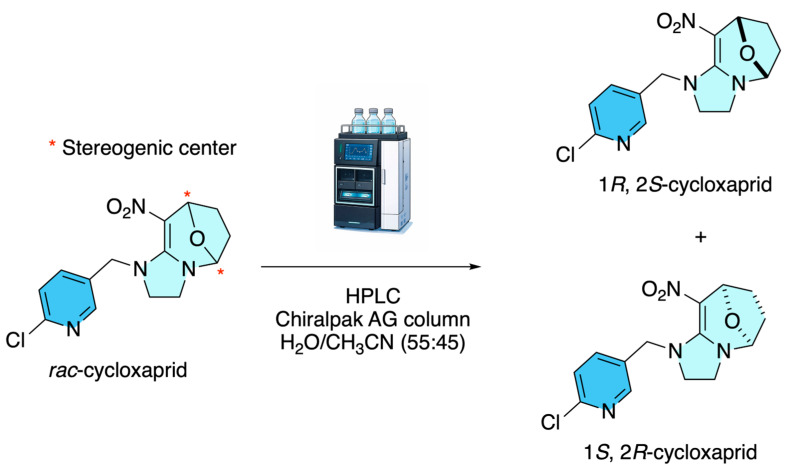
Chiral HPLC resolution of cycloxaprid.

In contrast, Wu and co-workers in 2016 reported the enantiomeric resolution of racemic cycloxaprid via semipreparative high-performance liquid chromatography using polysaccharide-based chiral stationary phases, specifically amylose tris(3-chloro-5-methylphenylcarbamate) (e.g., Chiralpak AG columns) [8]. As shown in Figure 3, under reverse-phase conditions, employing a water/acetonitrile mixture (55:45, *v*/*v*) at a flow rate of approximately 1.0 mL·min^−1^, baseline separation of the 1*R*,2*S*- and 1*S*,2*R*-enantiomers was achieved, enabling their isolation in optically pure form. This resolution arises from differential noncovalent interactions, primarily hydrogen bonding, π–π stacking, and steric complementarity, between the chiral selector and each enantiomer.

### 3.3. Dinotefuran Derivatives

**Figure 4:**
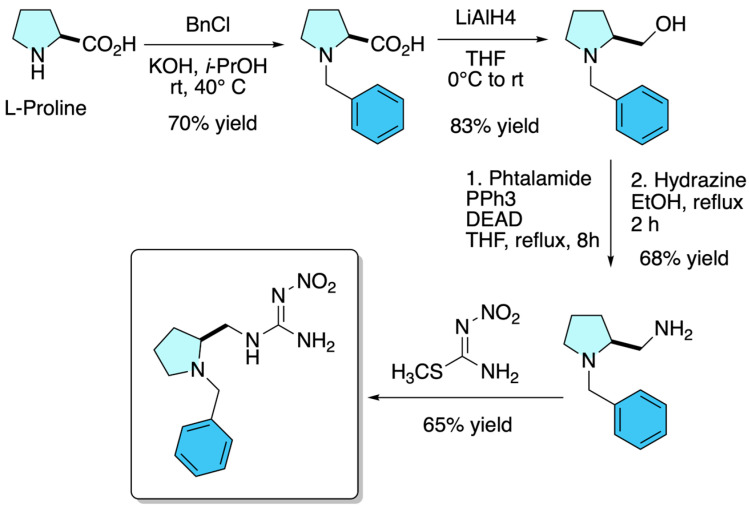
Synthetic route for the preparation of enantiopure L-proline-derived neonicotinoid analogues.

In a pioneering 2021 study, Olivares-Romero et al. reported the design and synthesis of a series of enantiopure neonicotinoid analogues derived from L-proline, using this natural amino acid as a predefined chiral scaffold to induce stereochemical control without the need for external asymmetric catalysis. As depicted in Figure 4, the synthetic strategy relied on the direct incorporation of L-proline into the nitroguanidine core, followed by sequential transformations including metal hydride reductions, Gabriel synthesis, and final couplings with nitroguanidine precursors, enabling the preparation of structurally diverse libraries with varying chain lengths and amine substitution patterns. Structural identification and characterization were carried out using ^1^H and ^13^C NMR spectroscopy, measurement of specific optical rotation as evidence of enantiopurity, and high-resolution mass spectrometry (HRMS-ESI) to confirm molecular formulas. Preservation of chirality throughout the synthetic route allowed a direct evaluation of the influence of absolute configuration on biological activity. In insecticidal assays against *Xyleborus affinis*, the L-proline-derived analogue exhibited higher activity than the commercial insecticide dinotefuran, together with a reduced neurotoxic profile in murine models [9].

**Figure 5:**
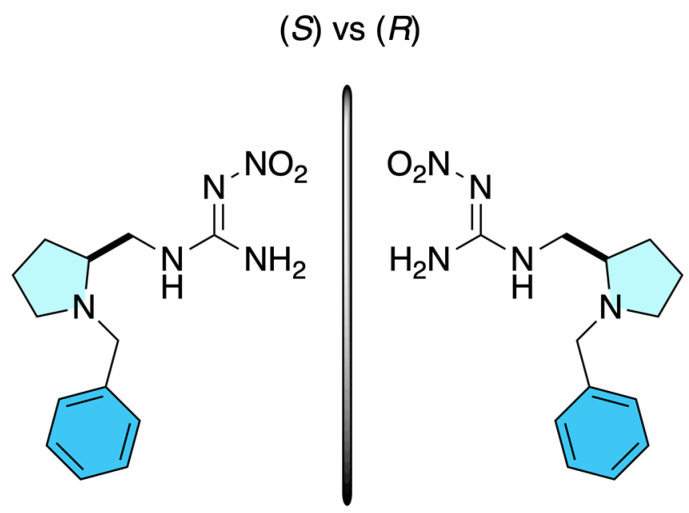
Chemical structures of the enantiomeric (*S*)-7 and (*R*)-7 proline-derived chiral neonicotinoid derivatives.

Dinotefuran-inspired chiral neonicotinoids were prepared enantioselectively through a chiral pool-based asymmetric strategy reported by Olivares-Romero et al. in 2024 [10] using (*R*)- and (*S*)-proline-derived amines as the source of molecular chirality. In this approach, the key step involves the incorporation of proline-based chiral amines into neonicotinoid frameworks structurally inspired by dinotefuran, enabling the direct synthesis of enantiomerically pure derivatives without the formation of racemic intermediates. The stereochemical information introduced from the starting chiral amino acid was preserved throughout the synthetic sequence, ensuring complete enantiocontrol without the need for post-synthetic resolution. Structural elucidation of the resulting enantiomers was achieved by ^1^H and ^13^C NMR spectroscopy, supported by DEPTQ-135 experiments and high-resolution mass spectrometry (HRMS-QTOF). Biological evaluation revealed pronounced enantioselectivity, with clear differences in insecticidal activity, toxicity, and environmental behavior between the (*R*)- and (*S*)-configured derivatives (Figure 5).

**Figure 6:**
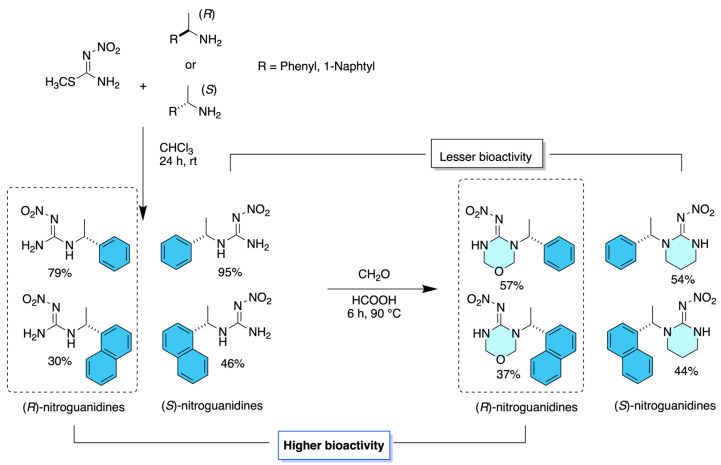
Synthesis of the amine-based diversity substituents and stereochemical configurations evaluated to assess structure–activity relationships.

Dinotefuran-related neonicotinoid derivatives were synthesized through a stepwise synthetic route based on chiral amine functionalization, as reported by Olivares-Romero et al. in 2021 [11]. The synthetic strategy relied on the structural modification of neonicotinoid cores using diverse amine substituents, including chiral amines, to generate a library of analogues without employing asymmetric catalysis or post-synthetic enantioseparation. Chirality in selected compounds was introduced by enantiomerically defined amines, allowing the preparation of derivatives with assigned absolute configuration. The synthesized compounds were structurally identified and characterized by comprehensive spectroscopic analysis, including ^1^H and ^13^C NMR spectroscopy for structural confirmation and high-resolution mass spectrometry (HRMS) to verify molecular formulas. Biological assays revealed that a subset of compounds exhibited enhanced insecticidal activity relative to other analogues within the series. Notably, the derivatives with *R* configuration were identified as the compounds with the most favorable biological performance, highlighting the influence of amine structure and stereochemical configuration on the insecticidal efficacy of dinotefuran-inspired neonicotinoids (Figure 6).

## 4. Chiral Pyrethroids

### 4.1. Cypermethrin and Cypermethrin Analogues

**Figure 7:**
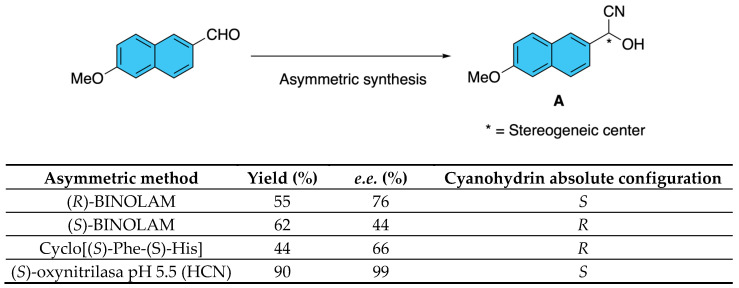
Enantioselective methods to prepare the cyanohydrin.

**Figure 8:**
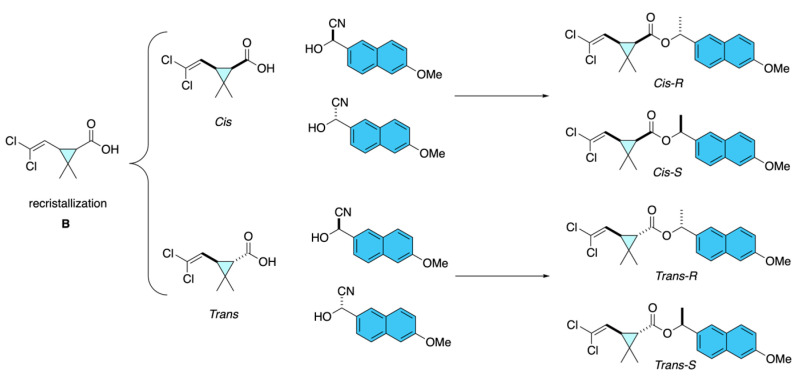
Asymmetric synthesis of cypermethrin analogues.

To obtain optically pure cypermethrin analogues, Huang and co-workers first synthesized enantioenriched cyanohydrins [(*R*)- or (*S*)-2-hydroxy-2-(6-methoxy-2-naphthyl)acetonitrile] through asymmetric cyanation of 6-methoxy-2-naphthaldehyde [12]. Among the methods evaluated, which include Lewis acid catalysis, organocatalysis, and biocatalysis, the most efficient method was the use of (*S*)-oxynitrilases from *Prunus amygdalus* or *Manihot esculenta*, which afforded cyanohydrins **A** with enantiomeric excesses exceeding 90% (Figure 7). These intermediates were subsequently esterified with optically pure *cis* or *trans* 3-(2,2-dichlorovinyl)-2,2-dimethylcyclopropane-1-carboxylic acid **B**, allowing the preparation of the individual *cis*- and *trans*-stereoisomers of cypermethrin fluorescent analogues. The products were isolated with high optical purity and structurally confirmed by chiral HPLC, GC/MS, NMR, and X-ray crystallography, thereby providing well-defined stereoisomers suitable for enzymatic and toxicological evaluation (Figure 8).

**Figure 9:**
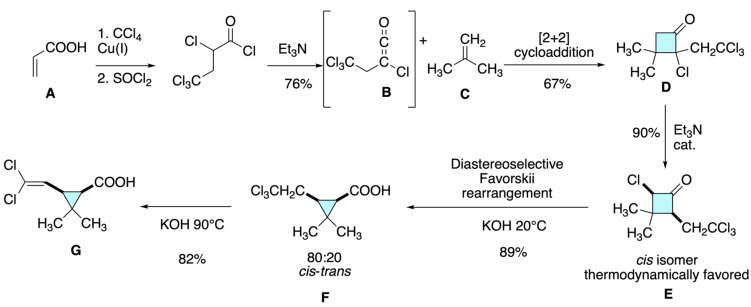
Stereoselective synthesis of a halovinylcyclopropanecarboxylic acid via copper(I)-catalyzed addition and Favorskii rearrangement, favoring the biologically active *cis* isomer.

Martin, Greuter, and Belluš reported a short and efficient synthesis of 2,2-dimethyl-3-(2,2-dichlorovinyl)cyclopropane-1-carboxylic acid, the key acid moiety of modern pyrethroids such as permethrin and cypermethrin [13]. As shown in Figure 9, their approach involved the copper(I)-catalyzed addition of carbon tetrachloride to acrylic acid **A**, followed by conversion to ketene **B** and a [2+2] cycloaddition with isobutylene **C** to afford a 2-chlorocyclobutanone intermediate **D**. A novel triethylamine-catalyzed isomerization furnished the thermodynamically favored 2,4-*cis* isomer **E**, which underwent Favorskii rearrangement to give the target acid **F** in good overall yield and with an 80:20 preference for the biologically active *cis* isomer. This strategy offered a stereoselective and versatile entry to halovinylcyclopropanecarboxylic acids **G**, overcoming the limitations of previous multi-step or hazardous methods.

**Figure 10:**
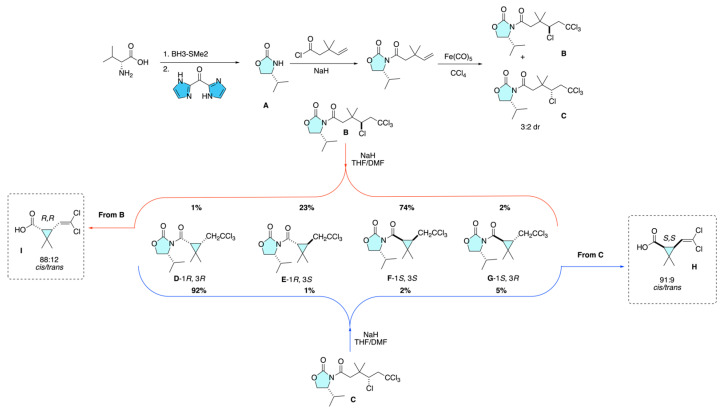
Evans auxiliary–directed enantioselective intramolecular cyclopropanation showing matched/mismatched enolate control and preferential *cis* isomer formation.

Kleschick and co-workers rendered the intramolecular alkylation approach enantioselective by employing a chiral Evans oxazolidinone auxiliary derived from (*R*)-valine to control both enolate facial selectivity and cyclopropanation outcome [14]. The auxiliary **A** was acylated and then subjected to Fe(CO)_5_/CCl_4_ addition to give tetrachloroacyl adducts **B** and **C** (3:2), which were separated by preparative HPLC. Base-promoted cyclization (NaH, THF/DMF) of **C**, where auxiliary bias and the preferred (*Z*)-enolate closure are matched, afforded a 92:1:2:5 mixture of cyclized products (**D**–**G**), dominated by the *cis* isomer; the mismatched isomer **B** gave 1:23:74:2. Subsequent methanolysis (LiOMe) and hydrolysis/dehydrochlorination (KOH) furnished the target acid **H** in 77% yield, with *cis*/*trans* 91:9 (from **C**) and acid **I**, 88:12 (from **B**); optical rotations corroborated formation of optically active (1*R*,3*R*)-I. These data support a model in which chiral enolate geometry and backside SN_2_-type ring closure combine to deliver high *cis* stereoselectivity (Figure 10).

**Figure 11:**
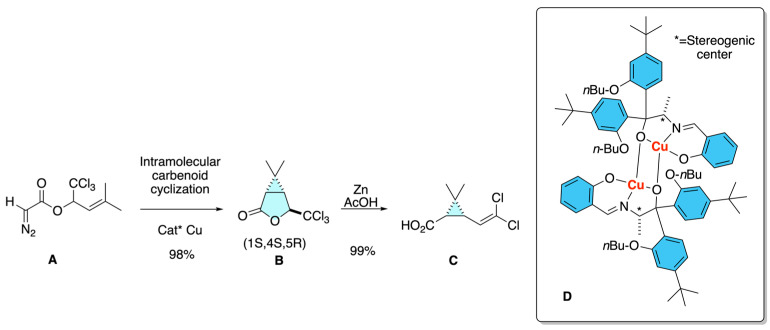
Intramolecular carbenoid cyclization of diazoacetoacetates as an early asymmetric approach to dimethylcyclopropanecarboxylic acids, key acid moieties of cypermethrin.

Kondo and co-workers patented a stereoselective route to synthesize *cis*-3-(2,2-dihalovinyl)-2,2-dimethylcyclopropanecarboxylic acids **C**, the acid moiety of cypermethrin [15]. Their strategy relied on stereoselective intramolecular carbenoid cyclization of diazoacetoacetates **A** to form bicyclic lactones **B** using the chiral Lewis acid **D**, followed by transformations such as Clemensen reduction to furnish the *cis*-cyclopropane framework **C**. Notably, they proposed obtaining enantioenriched products either from optically pure starting materials or by employing chiral copper catalysts during the cyclization, thereby constituting an early example of asymmetric synthesis applied to pyrethroid precursors (Figure 11).

**Figure 12:**
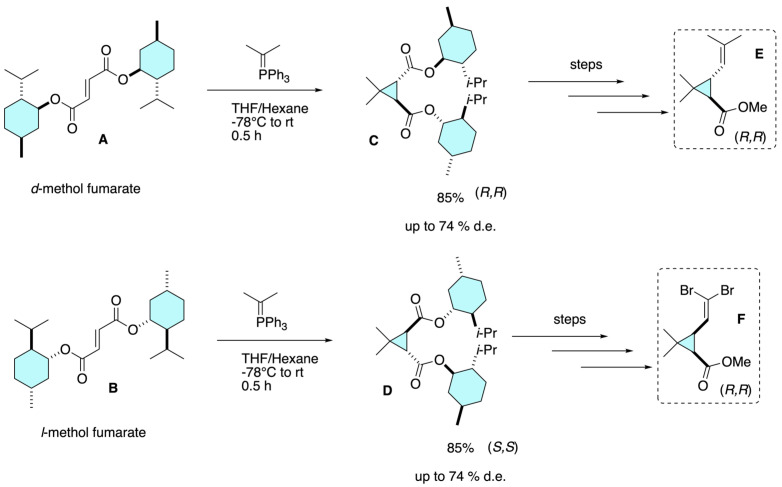
Early asymmetric synthesis of pyrethroid acid precursors via chiral auxiliary–controlled cyclopropanation of dimethyl fumarates.

In their seminal work, De Vos and Krief reported one of the first examples of an enantioselective synthesis of pyrethroid precursors relevant to cypermethrin analogs [16]. The strategy centered on the preparation of (1*R*)-*trans*-chrysanthemic ester **E** and its (1*R*)-*cis*-dibromovinyl analogue **F**, the stereogenic acid fragments incorporated in highly active pyrethroids. As depicted in Figure 12, the key step involved the use of dimethyl fumarates **A** or **B** as chiral auxiliaries, which underwent cyclopropanation with isopropylidene triphenylphosphorane to furnish carboxylic diesters **C** and **D** in high yield (85%) and with significant diastereoselectivity (up to 74% d.e.). Subsequent transesterification and selective saponification provided enantiomerically enriched caronic acid derivatives **E** and **F**, which were then elaborated to the corresponding chrysanthemic esters. By applying this sequence, the authors achieved access to optically pure acid building blocks that could be esterified with α-cyano-3-phenoxybenzyl alcohol to yield enantioenriched cypermethrin-type pyrethroids. This pioneering asymmetric induction approach demonstrated the feasibility of constructing the cyclopropane core of pyrethroids in a stereocontrolled fashion, thereby laying the groundwork for the development of commercial single-enantiomer formulations such as esfenvalerate and stereochemically defined cypermethrin analogs.

**Figure 13:**
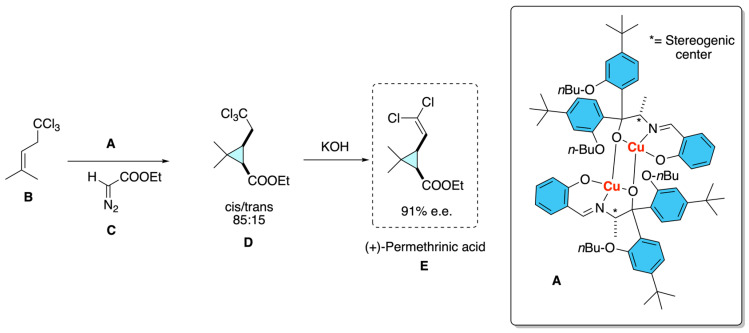
Enantioselective carbenoid cyclization.

In this classic contribution, Aratani demonstrated the catalytic asymmetric synthesis of cyclopropane carboxylic acids, which are the acid fragments of pyrethroid insecticides such as cypermethrin and permethrin [17]. As shown in Figure 13, their approach relied on the use of chiral copper–Schiff base complexes **A** derived from optically active α-amino acids and bulky aryl Grignard reagents to catalyze the cyclopropanation of olefins **B** with alkyl diazoacetates **C**. By fine-tuning the steric environment of the copper complex, they achieved high enantioselectivities, with enantiomeric excesses exceeding 90% for the formation of chrysanthemic acid derivatives **D**. Importantly, this methodology was extended to the synthesis of permethrinic acid **E**, the cyclopropane acid component of cypermethrin, where the optimized catalyst furnished the (+)-*cis* isomer with a *cis*/*trans* ratio of 85:15 and an *e.e.* of 91%.

This work also provided one of the earliest catalytic asymmetric routes to the stereodefined cyclopropane carboxylic acids essential for the preparation of highly active pyrethroid insecticides, directly linking stereocontrolled organometallic methodology to industrially relevant agrochemicals.

**Figure 14:**
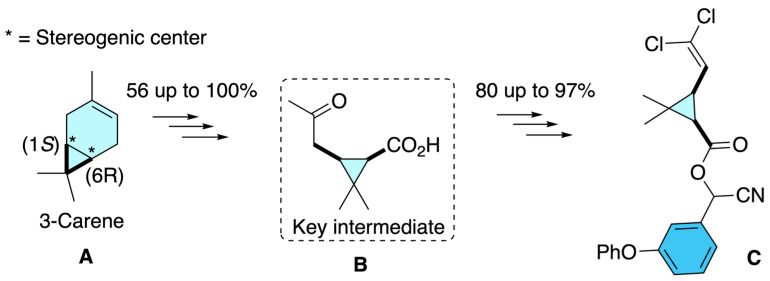
Terpene-based asymmetric approach to *cis*-configured pyrethroid intermediates using (+)-3-carene as a chiral pool precursor.

As depicted in Figure 14, Mandal and co-workers reported a novel asymmetric route to optically active pyrethroids, including cypermethrin, by exploiting the natural chirality of (+)-3-carene as a starting material [18]. Their approach began with the conversion of 3-carene **A** into functionalized bicyclic intermediates via epoxidation, rearrangement, and Baeyer–Villiger oxidation, yielding key *cis*-configured cyclopropane carboxylic acid derivatives **B**. These intermediates, inherently chiral due to the stereogenic centers derived from 3-carene, were further elaborated into the target insecticidal esters. For cypermethrin, the strategy involved esterification with 3-phenoxybenzyl alcohol and subsequent transformations introducing the dihalovinyl group, culminating in the preparation of (1*R*)-*cis*-(+)-cypermethrin **C** in optically enriched form. This route was significant because it avoided classical resolution techniques and instead used the chirality embedded in a natural terpene to achieve enantioenriched pyrethroids, thus demonstrating a scalable and conceptually elegant approach to stereodefined insecticides.

**Figure 15:**
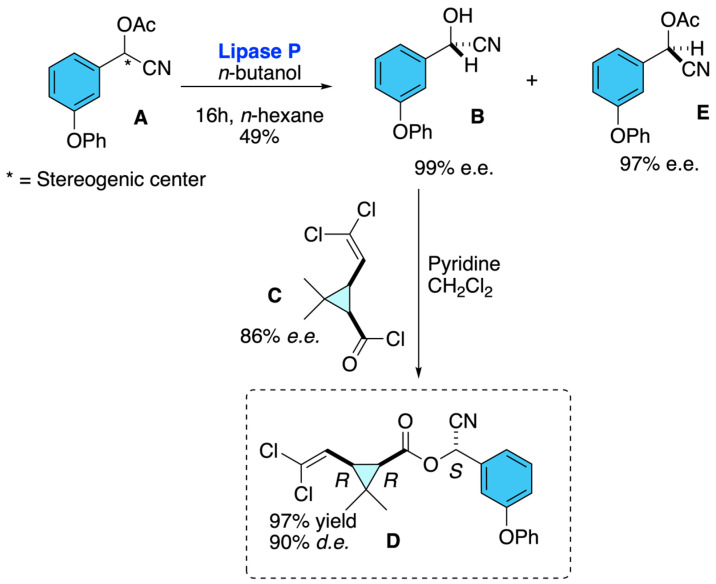
Lipase-mediated kinetic resolution strategy for the preparation of enantiopure (1*R*,*cis*,α*S*)-cypermethrin.

Roos, Stelzer, and Effenberger developed a practical procedure for obtaining enantiopure (1*R*,*cis*,α*S*)-cypermethrin, the stereoisomer with the highest biological activity [19]. Their strategy relied on the enzymatic kinetic resolution of racemic m-phenoxybenzaldehyde cyanohydrin acetate **A** using immobilized lipase P, which catalyzed the transesterification with *n*-butanol to afford the (*S*)-cyanohydrin **B** with >99% *e*.*e*. This enantiomerically enriched intermediate was then directly acylated with the (1*R*,*cis*)-cyclopropanecarboxylic acid chloride **C**, producing (1*R*,*cis*,α*S*)-cypermethrin **D** with an optical purity of 99% *d.e*. The unreacted (*R*)-enantiomer **E** was recovered by distillation and racemized with triethylamine, allowing it to be recycled into the process, thereby establishing a continuous and efficient cycle. Overall, the method delivered the desired enantiopure insecticide in 80% total yield, providing a robust biocatalytic approach for the large-scale preparation of stereodefined pyrethroid (Figure 15).

**Figure 16:**
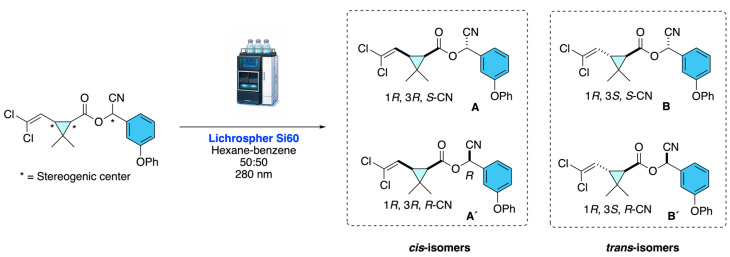
HPLC-based resolution of cypermethrin stereoisomers combining UV and polarimetric detection.

Navas-Díaz and Co-workers [20] describe that Cypermethrin stereoisomers were resolved by normal-phase HPLC on a Lichrospher Si60 column using hexane–benzene (50:50) as the mobile phase, with UV (280 nm) and diode-laser polarimetric detection in series. The method enabled the separation of *cis*- and *trans*-diastereomers by UV and the direct discrimination of the four enantiomers by polarimetry (Figure 16).

**Figure 17:**
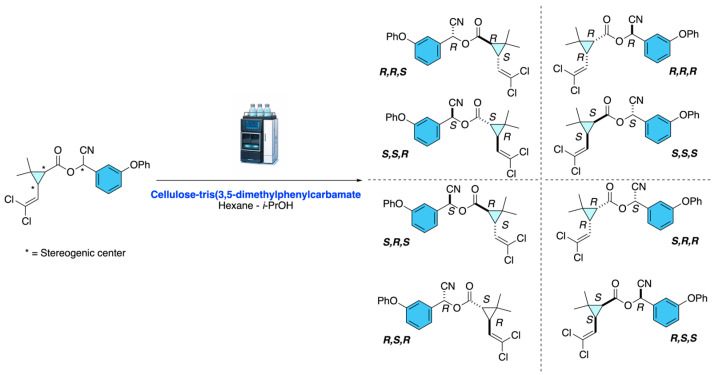
HPLC resolution of cypermethrin stereoisomers.

As depicted in Figure 17, cypermethrin stereoisomers were extracted by QuEChERS (Quick, Easy, Cheap, Effective, Rugged, and Safe) or QuOil (a QuEChERS modification for high-oil matrices), followed by d-SPE cleanup, and analyzed using LC–MS/MS on a Waters Cortecs UPLC C18 column with ESI+ detection. The method resolved the four enantiomeric pairs into distinct peaks, using a cellulose-tris(3,5-dimethylphenylcarbamate) stationary phase, and n-hexane/i-propanol as modifier. The method enables reliable quantification of cypermethrin and α-cypermethrin in diverse food matrices, while avoiding isomerization artifacts common in GC analysis [21,22].

### 4.2. Fenvalerate and Fenvalerate Analogues

**Figure 18:**
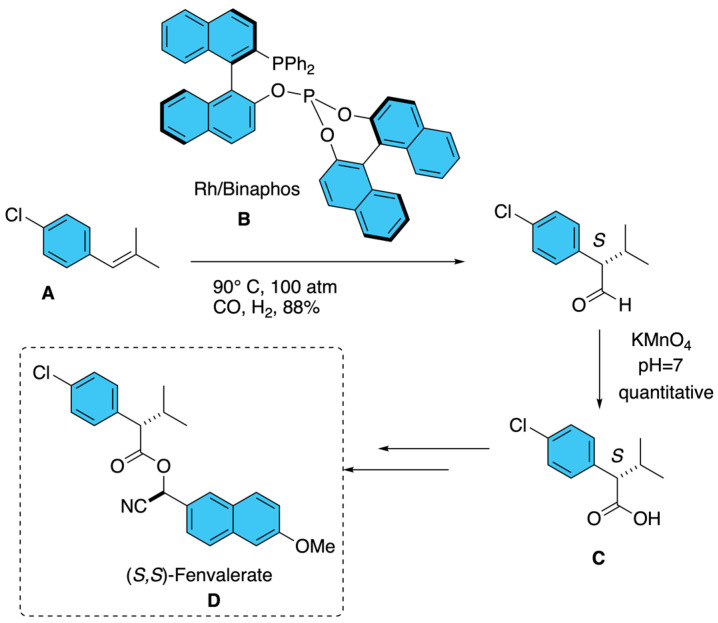
Enantioselective hydroformylation catalyzed by Rh-Binaphos.

Fenvalerate is a non-cyclopropane synthetic pyrethroid characterized by the presence of two stereogenic centers. Among the four possible stereoisomers, the diastereomer in which both centers adopt the (*S*)-configuration displays the highest insecticidal potency. The optically active form corresponding to this configuration is commercially known as esfenvalerate. In this context, as shown in Figure 18, the enantioselective hydroformylation of 1-chloro-4-(2-methylprop-1-enyl)benzene **A**, catalyzed by rhodium carbonyl complexes **B**, offers a viable strategy for accessing (*S*)-2-(4-chlorophenyl)-3-methylbutanoic acid **C**, a key precursor in the synthesis of fenvalerate **D** [23].

**Figure 19:**
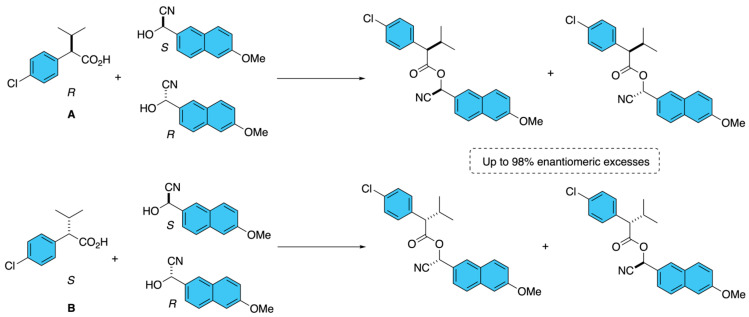
Enantioselective synthesis and stereochemical assignment of fenvalerate analogues using enantiopure cyanohydrin building blocks.

Another example of fenvalerate analogues synthesis followed a similar approach to that of the cypermethrin analogues. The optically pure cyanohydrins also served as chiral building blocks for esterification with the enantiomers (*R*) or (*S*) of 2-(4-chlorophenyl)-3-methyl butanoic acid, **A** and **B**, respectively (Figure 19) [12]. This route enabled access to the four distinct stereoisomers of fenvalerate fluorescent analogues. Chiral separation and stereochemical assignment were performed using a combination of chiral HPLC and NMR analysis, supported by crystallographic data. The resulting compounds exhibited enantiomeric excess values above 98%, demonstrating the effectiveness of the combined catalytic and enzymatic strategies for generating highly enantioenriched fenvalerate analogues. These optically defined products were subsequently used to investigate the stereoselective hydrolysis of fenvalerate by murine carboxylesterases.

**Figure 20:**
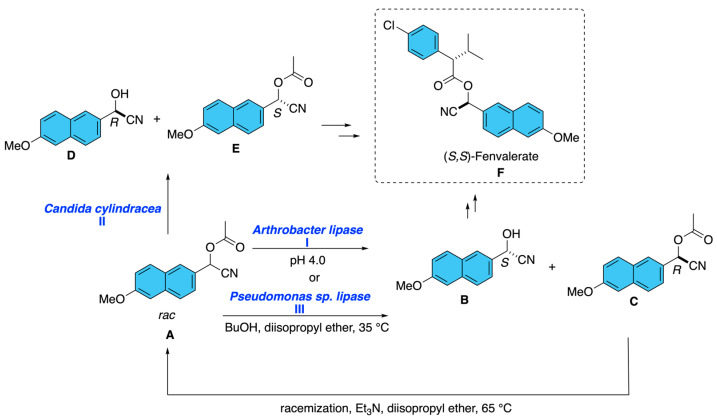
Enzymatic kinetic resolution of racemic α-cyano-3-phenoxybenzyl acetate.

A recurrent stereoselective strategy toward fenvalerate-type pyrethroids has been the enzymatic kinetic resolution of racemic cyano(6-methoxynapthalen-2-yl)methyl acetate **A**, a direct precursor of the biologically relevant alcohol moiety. Mitsuda and co-workers showed that several microbial lipases preferentially hydrolyze the (*S*)-acetate, but *Arthrobacter lipase* was especially effective, affording optically pure (*S*)-2-hydroxy-2-(6-methoxynapthalen-2yl)acetonitrile **B** under mildly acidic conditions (pH 4.0), while the unreacted (*R*)-acetate **C** could be racemized and recycled (Figure 20, I) [24]. Another process reported the isolation of the protected (*S*)-cyano(6-methoxynapthalen-2-yl)methyl acetate itself. The disclosed method used microbial lipase, particularly *Candida cylindracea* lipase [25], to asymmetrically hydrolyze the (*R*) ester from racemic acetate **A**, leaving the (*S*) acetate **E** enriched and recoverable after separation from the corresponding alcohol **D**. Conceptually, this process is notable because it targets the stereodefined intermediates, which are flexible synthons for the subsequent assembly of fenvalerate (Figure 20, II). A more process-oriented advance was reported by Fishman and Zviely [26], who designed a scalable chemoenzymatic route to (*S*)-2-hydroxy-2-(6-methoxynaphthalen-2-yl)acetonitrile **B** beginning from racemic acetate **A**. Their sequence comprised the transformation of racemic cyano(6-methoxynapthalen-2-yl)methyl acetate **A** via *Pseudomonas* sp. lipase-mediated transesterification in organic solvent, followed by the separation of the enantiopure alcohol **B**, and racemization of the undesired ester **C** for recycling. Importantly, the use of organic medium minimized cyanohydrin decomposition and product racemization, while immobilized enzyme operation enabled both batch reuse and continuous processing (Figure 20, III).

### 4.3. Cycloprothrin and Cycloprothrin Analogues

**Figure 21:**
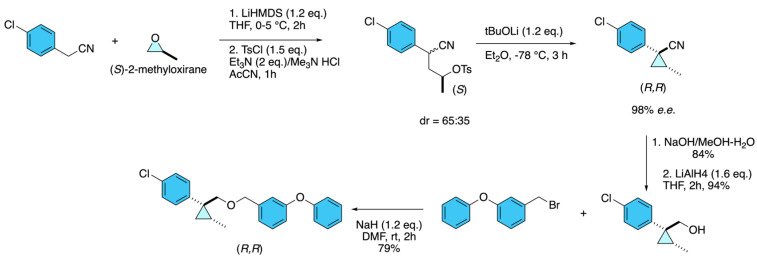
Asymmetric cyclopropanation strategy for the synthesis of optically pure cycloprothrin analogues.

Taniguchi and co-workers [27] reported the asymmetric synthesis of cycloprothrin analogues by constructing cyclopropane pyrethroids bearing two stereogenic centers on the cyclopropane ring. The key step involved the enantioselective ring-opening of chiral propylene oxides with 4-chlorobenzyl cyanide anion, followed by tosylation and an S_N_2-type cyclopropanation. Using (*R*)- and (*S*)-propylene oxide, the authors accessed enantioenriched cyclopropane nitrile precursors with excellent optical purity (>98% *e.e.*, confirmed by chiral HPLC). These intermediates were transformed into cyanohydrin esters and ether derivatives, enabling direct evaluation of structure–activity relationships. Biological assays against *Culex pipiens pallens* revealed clear chiral discrimination, as only the (1*R*,2*R*)-configured ether showed strong insecticidal activity, while the cyanohydrin esters were inactive. This work demonstrates how enantioselective cyclopropanation strategies can efficiently deliver optically pure cycloprothrin analogues, highlighting the critical role of stereochemistry in pyrethroid insecticidal activity (Figure 21).

**Figure 22:**
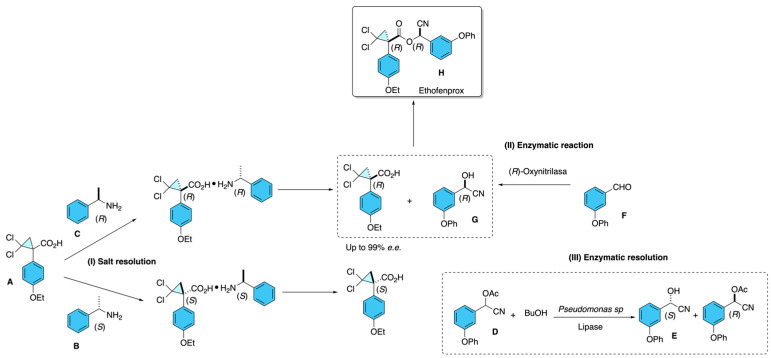
Diastereomeric salt separation, enzymatic reaction, and enzymatic resolution.

As depicted in Figure 22, Jiang and Co-workers [28,29] employed multiple asymmetric synthesis and resolution strategies to obtain optically active isomers of ethofenprox, demonstrating a deliberate application of chiral tools to enhance insecticidal activity. They first resolved the racemic acid intermediate, 1-(4-ethoxyphenyl)-2,2-dichlorocyclopropane-1-carboxylic acid **A**, using chiral amines such as *S*-(−)-α-methylbenzylamine or *R*-(+)-α-methylbenzylamine **B** or **C**, respectively, to form diastereomeric salts, which were then crystallized and acidified to yield enantiopure acids with high enantiomeric excess (*e.e.* ≈ 99.8%) [Figure 22, incise (I)]. For the alcohol segment, 2-hydroxy-2-(3-phenoxyphenyl)acetonitrile, they utilized both enzymatic and chemoenzymatic approaches: lipase from *Pseudomonas* sp. was used to kinetically resolve the racemic acetate **D** via transesterification to obtain the (*S*)-alcohol **E** [Figure 22, incise (III)], while an (*R*)-oxynitrilase extracted from bitter almonds catalyzed the enantioselective addition of hydrogen cyanide to 3-phenoxybenzaldehyde **F** to furnish the (*R*)-alcohol **G** [Figure 22, incise (II)]. Finally, these resolved chiral building blocks were coupled via esterification using condensing agents like DCC or EDCI, yielding a specific diastereomer of ethofenprox **H**. This strategic use of asymmetric synthesis and resolution enabled the authors to isolate isomers such as the highly active (*R*,*R*)-isomer, which showed significantly improved insecticidal potency compared to the racemic mixture [28,29].

**Figure 23:**
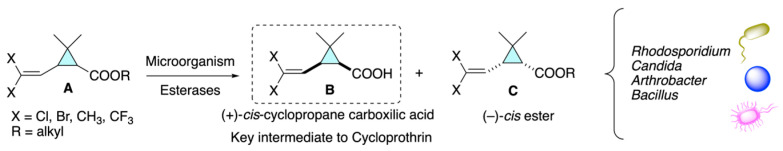
Biocatalytic asymmetric hydrolysis of cyclopropanecarboxylic acid esters for the preparation of optically active pyrethroid intermediates.

Nishizama and Co-workers [30] developed an enzymatic asymmetric hydrolysis process to obtain optically active cyclopropanecarboxylic acids, key intermediates for pyrethroid insecticides such as cycloprothrin. They utilized specific microorganisms or esterases derived therefrom, including strains from *Rhodosporidium*, *Candida*, *Arthrobacter*, *Bacillus*, and other genera, to selectively hydrolyze racemic *cis*-cyclopropanecarboxylic acid esters **A**. This biocatalytic approach preferentially cleaved the (+)-*cis* enantiomer **B**, leaving the (−)-*cis* ester **C** unchanged and enabling the isolation of the desired (+)-*cis* acid in high optical purity. The method offered significant improvements in yield and enantioselectivity over prior chemical or enzymatic methods, facilitating a more efficient and industrially viable route to these chiral building blocks (Figure 23).

## 5. Chiral Oxadiazines

### Indoxacarb and Indoxacarb Analogues

Indoxacarb is a broad-spectrum oxadiazine insecticide that has become an important member of modern agrochemical arsenals due to its unique mode of action and favorable selectivity profile. It acts primarily as a voltage-dependent sodium channel blocker, leading to paralysis and death in a wide range of lepidopteran and other insect pests, while exhibiting comparatively low toxicity to mammals and beneficial organisms. Structurally, indoxacarb is characterized by a stereogenic center within its indane-derived moiety, making its biological activity enantioselective―only the (−)-enantiomer demonstrates potent insecticidal properties. This stereochemical requirement distinguishes indoxacarb from many earlier insecticides, where racemic mixtures were commonly commercialized, and highlights the importance of asymmetric synthesis in its development. The discovery and commercialization of indoxacarb thus represent a milestone, as they exemplify how enantioselective synthetic strategies can be directly linked to enhanced efficacy and safety in insecticide design, establishing a paradigm for the preparation of future chiral agrochemicals [31].

**Figure 24:**
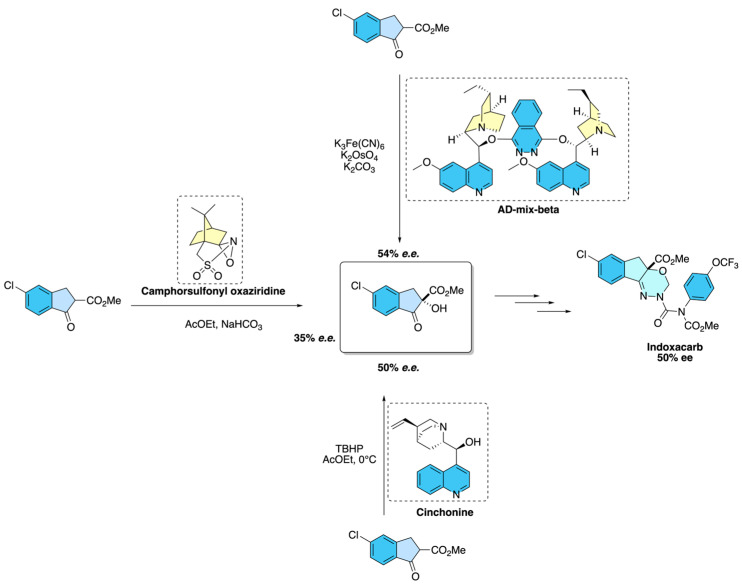
Representative asymmetric synthesis of Indoxacarb.

In this context, McCann and co-workers [32] demonstrated that asymmetric synthesis played a pivotal role in the development of indoxacarb, an oxadiazine insecticide, where only one enantiomer exhibited potent biological activity. The team introduced chirality at the stage of 2-carbomethoxyindanone hydroxylation, exploring several enantioselective methods. Sharpless asymmetric dihydroxylation provided hydroxyindanone intermediates with moderate enantioselectivity (~50% *e.e.*), while camphorsulfonyl oxaziridines gave slightly lower values. The most effective strategy employed a cinchonine-catalyzed hydroxylation with tert-butyl hydroperoxide, which reproducibly delivered the (−)-hydroxyindanone with ~50% *e.e.* on a preparative scale. Subsequent conversion of this intermediate through semicarbazone formation and *O*,*N*-acetal cyclization furnished the commercial product, (−)-indoxacarb, underscoring how asymmetric oxidation enabled the scalable preparation of a stereochemically defined agrochemical (Figure 24).

**Figure 25:**
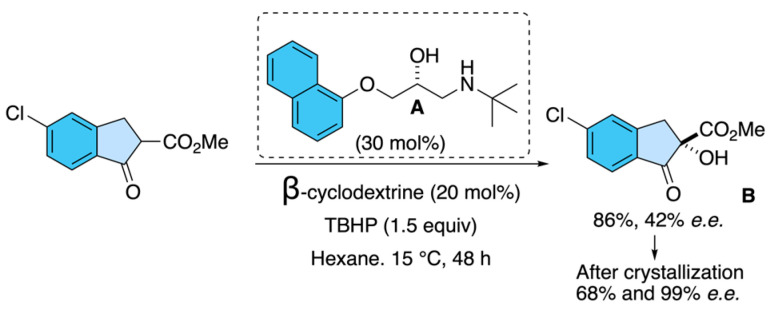
Organocatalytic asymmetric oxidation.

Gong and co-workers [33] reported an asymmetric synthetic strategy highly relevant for the preparation of indoxacarb’s chiral intermediates, specifically the α-hydroxylated indanone derivatives that serve as precursors to the insecticide. Their work employed commercially available chiral drugs as organocatalysts for the asymmetric α-hydroxylation of β-keto esters. Among the screened catalysts, timolol and propranolol showed promising enantioinduction (up to 32% and 18% *e.e.*, respectively), which inspired the design of twelve analogues. The optimized catalyst, (*R*)-1-(*tert*-butylamino)-3-(2-naphthoxy)-2-propanol **A**, in combination with β-cyclodextrin as cocatalyst and *tert*-butyl hydroperoxide as oxidant, delivered the desired (*S*)-hydroxyindanone ester **B** with up to 42% *e.e.* and 86% yield. Importantly, a single recrystallization step raised the optical purity to >99% *e*.*e*., with an isolated yield of 68% (Figure 25). This work illustrates how drug-inspired organocatalysts can provide a practical and scalable route to enantioenriched indanone building blocks, thus enabling the asymmetric synthesis of indoxacarb [28].

**Figure 26:**
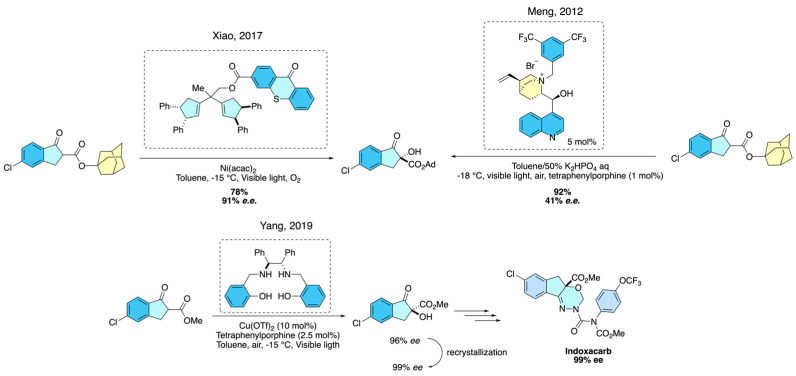
Photocatalytic and copper–salan-mediated enantioselective α-hydroxylation of β-keto esters toward chiral indoxacarb intermediates.

Other methodologies were reported by Lian and Ding [34,35] describing the α-hydroxylation of β-keto esters using phase transfer catalyst-visible light and bifunctional photocatalysis-Ni(II)-visible light, respectively. Subsequently, Yang and co-workers [36] introduced an innovative visible light-induced salan–copper(II)-catalyzed enantioselective α-hydroxylation of β-keto esters, a transformation directly relevant to the synthesis of indoxacarb’s chiral intermediates. Using tetraphenylporphyrin (TPP) as a photosensitizer to generate singlet oxygen from air, the system delivered α-hydroxy-β-keto esters in up to 95% yield and 96% *e.e.* under mild aerobic conditions. Importantly, substrates derived from indanone methyl esters, including 5-chloro-indanone esters, were efficiently hydroxylated to provide the (*S*)-configured intermediates, which, after straightforward derivatization, were converted into (*S*)-indoxacarb, the biologically active enantiomer. This methodology highlights how photoredox catalysis coupled with copper–salan complexes offers a sustainable and highly stereoselective approach to access key building blocks for enantiopure agrochemicals such as indoxacarb (Figure 26).

**Figure 27:**
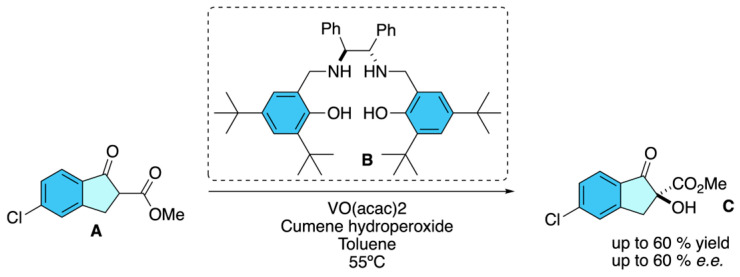
Asymmetric hydroxylation route to (*S*)-5-chloro-2-methoxycarbonyl-2-hydroxy-1-indanone using a chiral vanadyl–diamine catalyst, providing a key intermediate for the synthesis of biologically active (*S*)-indoxacarb.

Since 2013, several patents have progressively advanced the asymmetric synthesis of *S*-indoxacarb, focusing on the α-hydroxylation of 5-chloro-2-methoxycarbonyl-1-indanone, the key chiral intermediate. The earliest report by Sun [37] discloses an asymmetric hydroxylation route from ester **A** to the key intermediate (*S*)-5-chloro-2-methoxycarbonyl-2-hydroxy-1-indanone **C** for indoxacarb. Using a chiral vanadyl–diamine complex **B** with cumene hydroperoxide as oxidant, the method provided the desired (*S*)-indanone **C** in ~57–60% yield with about 60% *e.e.*, as confirmed by chiral HPLC analysis. Although the enantioselectivity is moderate, this process demonstrates a practical entry to the biologically active enantiomer of indoxacarb (Figure 27). More recently, Bo and Co-workers [38] refined these strategies by incorporating recyclable zirconium complexes that deliver consistently high optical purity and scalability, establishing a sustainable and cost-effective industrial route. Collectively, these inventions illustrate the evolution from moderate selectivity to robust, recyclable catalytic systems, cementing zirconium-based asymmetric hydroxylation as the current standard for large-scale production of enantiopure indoxacarb.

**Figure 28:**
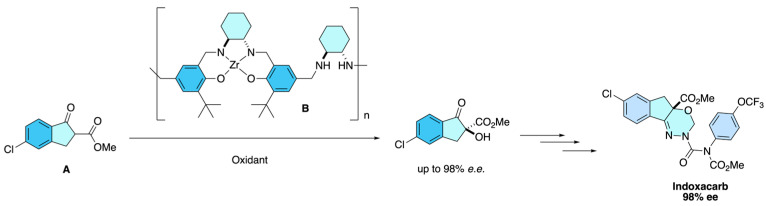
Zirconium-catalyzed asymmetric α-hydroxylation of indanone derivatives affording the key (*S*)-5-chloro-2-methoxycarbonyl-2-hydroxy-1-indanone intermediate with high enantioselectivity and catalyst recyclability for the synthesis of enantiopure (*S*)-indoxacarb.

This method employs a zirconium-based chiral catalyst system **B** combined with peroxides under mild conditions to achieve the α-hydroxylation of the indanone substrate **A**. Compared with earlier vanadium- or cinchonine-catalyzed oxidations, the process delivers both higher enantioselectivity (*e.e.* > 98%) and improved yields, while also enabling catalyst recovery and reuse of up to 10 cycles without a significant drop in either enantioselectivity or yield. These advances overcome the limitations of previous asymmetric hydroxylation strategies, providing a more practical and scalable synthesis suitable for the industrial production of enantiopure indoxacarb (Figure 28) [33].

**Figure 29:**
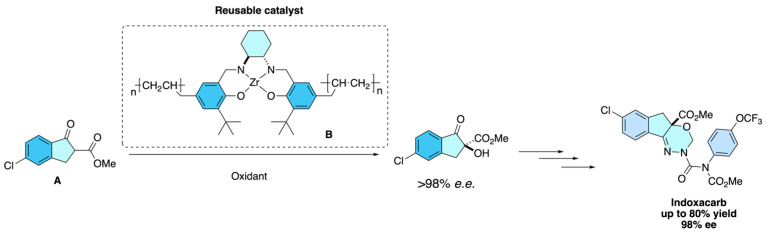
Optimized zirconium-catalyzed asymmetric hydroxylation of 5-chloro-2-methoxycarbonyl-1-indanone, enabling the efficient and recyclable synthesis of enantiopure (*S*)-indoxacarb.

The 2024 U.S. patent [39] describes a further optimized process for preparing *S*-indoxacarb via asymmetric hydroxylation of 5-chloro-2-methoxycarbonyl-1-indanone **A**. The method employs recoverable zirconium-based chiral catalysts **B**, which improve both efficiency and sustainability of the transformation. Compared to earlier strategies, this invention achieves higher enantiomeric purity (>98% *e.e.*) while ensuring the catalyst can be recycled multiple times without significant loss of performance, thereby reducing production costs and environmental impact (Figure 29).

**Figure 30:**
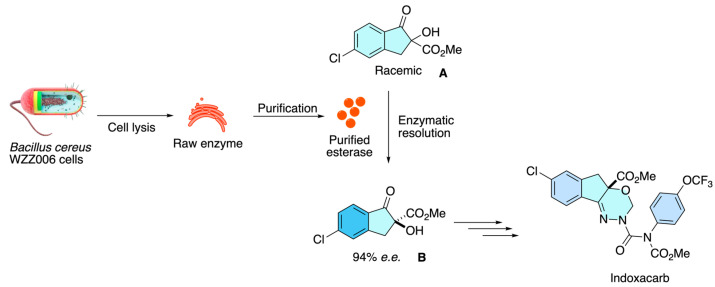
Enzymatic kinetic resolution of a racemic indoxacarb intermediate using a purified thermoalkaliphilic esterase from Bacillus cereus, enabling efficient access to the (*S*)-configured precursor under mild conditions.

Zhang and co-workers [40] report an enzymatic asymmetric route to indoxacarb via enantioselective resolution of its key intermediate, (*R*,*S*)-5-chloro-1-oxo-2,3-dihydro-2-hydroxy-1H-indene-2-carboxylic acid methyl ester **A**. They purified a thermoalkaliphilic intracellular esterase (BCE) from Bacillus cereus WZZ006 (96 kDa; 89.5-fold purification; specific activity 1.79 U mg^−1^), showing optimal activity at pH 8.5 and 50 °C. Using purified BCE, the kinetic resolution of racemic **A** achieved an enantiomeric excess of 94% at ~53% conversion in 3 h (E = 39.95), markedly shortening the process compared with whole-cell catalysis (36 h). Also, the performance was quantified by chiral HPLC and provided kinetic parameters (Km = 0.98 mM, kcat = 69.47 min^−1^), underscoring BCE as a practical biocatalyst to access the (*S*)-configured indoxacarb intermediate **B** under mild, industry-relevant conditions (Figure 30).

## 6. Comparative Evaluation of Strategies and General Principles for Enantioselective Synthesis of Insecticides, and Future Perspectives

**Figure 31:**
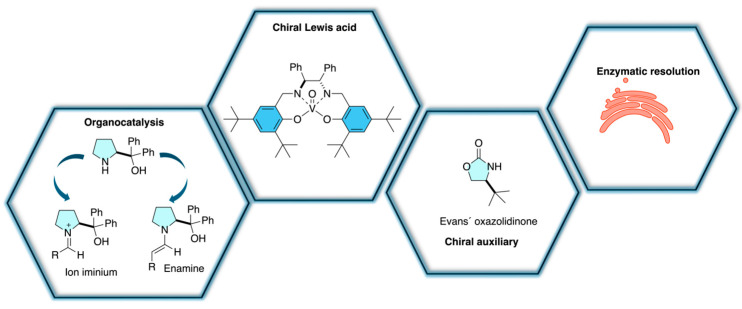
Stereoselective methodologies.

A broad range of methodologies has been developed for the enantioselective synthesis of insecticides, including organocatalysis, transition metal catalysis, chiral pool synthesis, biocatalysis, and classical resolution techniques, each with distinct advantages and limitations in terms of efficiency, scalability, and sustainability. Organocatalytic approaches provide operational simplicity and metal-free conditions, whereas transition metal catalysis generally affords higher and more consistent enantioselectivities suitable for industrial application. Chiral pool strategies offer straightforward access to enantiopure products from naturally available precursors but are restricted by substrate diversity. In contrast, biocatalytic methods deliver excellent stereoselectivity under mild and environmentally benign conditions, although enzyme stability and substrate scope may limit broader applicability. Classical resolution methods remain useful for analytical and small-scale purposes but are less attractive for industrial production due to material loss and additional processing requirements (Table 1). Despite mechanistic differences, these methodologies share common principles of stereocontrol, where chiral environments precisely organize reactive intermediates through steric, electronic, or coordination effects to favor selective bond formation (Figure 31).

Future advances in asymmetric insecticide synthesis will depend on integrating efficient catalytic systems with sustainable and scalable technologies. Current priorities include the development of organocatalytic, metal-based, and enzymatic platforms capable of achieving high enantioselectivity under industrially viable conditions, together with the implementation of green chemistry strategies such as continuous-flow and mechanochemical synthesis. Equally important is improving the understanding of the relationship between stereochemistry and biological activity through combined synthetic, toxicological, and environmental studies. In this context, computational methods, including DFT calculations, molecular modeling, and machine learning, are expected to accelerate catalyst optimization and enantioselectivity prediction. Overall, the transition from racemic mixtures to single-enantiomer insecticides is likely to become increasingly important for both regulatory compliance and the development of safer, more selective agrochemicals.

## 7. Stereochemistry–Activity Relationships in Chiral Insecticides

The biological activity of chiral insecticides is highly dependent on absolute configuration, as enantiomers interact differently with targets such as nicotinic acetylcholine receptors and voltage-gated sodium channels. Typically, only one stereoisomer provides the desired insecticidal effect, whereas others may show low activity or increased toxicity toward non-target organisms. In neonicotinoids, stereochemical constraints in compounds such as cycloxaprid, paichongding, and dinotefuran analogues enhance receptor binding, potency, and selectivity by favoring optimal conformations. Pyrethroids provide another clear example of stereochemistry-dependent activity, where only specific stereoisomers, such as the (1*R*,*cis*,α*S*)-isomer of cypermethrin, exhibit high insecticidal potency, leading to the development of enriched single-isomer formulations. Similarly, in indoxacarb and cycloprothrin analogues, only selected enantiomers display strong biological activity. Collectively, these studies demonstrate that stereochemistry and conformational control are critical determinants of efficacy, selectivity, and environmental safety, emphasizing the importance of asymmetric synthesis and stereochemical design in next-generation insecticides.

## 8. Conclusions

The analysis presented in this review underscores the pivotal role of stereochemistry in shaping the next generation of insecticides. Across diverse chemical classes, including neonicotinoids, pyrethroids, and oxadiazines, enantioselectivity has been consistently shown to influence not only insecticidal potency but also environmental fate, selectivity toward non-target organisms, and resistance profiles. These findings reinforce the concept that enantiomers should be regarded as distinct functional entities rather than interchangeable forms of the same compound. Significant progress has been achieved in the development of stereoselective synthetic methodologies. Organocatalysis, transition metal catalysis, chiral pool strategies, and biocatalysis have each contributed valuable tools for accessing enantiomerically enriched insecticides. In particular, advances in photochemical activation, enzyme engineering, and recyclable catalytic systems have improved both efficiency and sustainability, while recent industrial patents demonstrate the feasibility of scaling asymmetric processes to meet commercial demands. Nevertheless, no single strategy has emerged as universally applicable, and many methods remain limited by cost, operational complexity, or substrate scope. A critical gap identified in the current literature is the insufficient integration of synthetic methodologies with structure–activity relationships and environmental impact studies. While numerous reports describe the preparation of chiral insecticides, fewer systematically correlate absolute configuration with biological performance, toxicity, and degradation behavior. Addressing this disconnect will be essential for the rational design of safer and more effective agrochemicals. Looking forward, the field is poised to benefit from the convergence of synthetic chemistry with emerging technologies. The application of computational chemistry, molecular docking, and machine learning offers new opportunities to predict enantioselectivity and optimize catalyst design. In parallel, the adoption of green chemistry principles, including solvent minimization, catalytic efficiency, and energy-efficient processes such as flow and mechanochemistry, will be crucial for developing sustainable manufacturing routes. In conclusion, the transition from racemic mixtures to enantiomerically pure insecticides represents both a scientific challenge and an opportunity for innovation. Continued interdisciplinary efforts will be required to bridge the gap between laboratory-scale discovery and industrial implementation, ultimately enabling the development of next-generation agrochemicals that combine high efficacy with reduced environmental impact.

## Figures and Tables

**Table 1:** Comparative evaluation of strategies.

Strategy	Typical *e.e.* (%)	Yield	Scalability	Advantages	Limitations
Organocatalysis	70–95	Moderate–High	Moderate	Metal-free, mild conditions	Limited scalability, catalyst loading
Metal catalysis	85–99	High	High	High efficiency, industrial relevance	Metal cost, toxicity concerns
Chiral pool	>99	High	High	Simple, predictable stereochemistry	Limited substrate diversity
Biocatalysis	90–99	High	High	High selectivity, green conditions	Narrow substrate scope
Resolution (HPLC/salts)	>99	Low–Moderate	Low	High purity	Wasteful, not scalable

## Data Availability

No new data were created or analyzed in this study.

## Abbreviations

The following abbreviations are used in this manuscript:

nAChRs	Nicotinic Acetyl Choline Receptors
EFSA	European Food Safety Authority
HPLC	High Pressure Liquid Chromatography
NMR	Nuclear Magnetic Resonance
*e.e.*	Enantiomeric excess
*d.e.*	Diastereomeric excess
LC-MS	Liquid Chromatography–Mass Spectrometry
GC	Gas Chromatography
MS	Mass Spectrometry
DEPTQ-135	Distortionless Enhancement by Polarization Transfer with a 135-degree pulse angle
HRMS-QTOF	High Resolution Mass Spectrometry-Quadrupole Time of Flight
UPLC	Ultra-Performance Liquid Chromatography
C18	Octadecilsylane column chromatography
QuEChERS	Quick, Easy, Cheap, Effective, Rugged, and Safe extraction
QuOil	QuEChERS modification for high-oil matrices
DCC	*N*,*N’*-diciclohexylcarbodiimide
EDCI	1-Ethyl-3-(3-dimethylaminopropyl)carbodiimide
TPP	Tetraphenylporphyrin
BCE	Bacillus cereus WZ2006
